# Usefulness of an additional lead shielding device in reducing occupational radiation exposure during interventional endoscopic procedures: An observational study

**DOI:** 10.1097/MD.0000000000021831

**Published:** 2020-08-21

**Authors:** Reiko Yamada, Yusuke Saimyo, Kyosuke Tanaka, Aiji Hattori, Yuhei Umeda, Naoki Kuroda, Junya Tsuboi, Yasuhiko Hamada, Yoshiyuki Takei

**Affiliations:** a Department of Gastroenterology and Hepatology; b Department of Clinical Engineering, Ise Red Cross Hospital, Ise; c Department of Endoscopy, Mie University Hospital, Tsu, Mie, Japan.

**Keywords:** endoscopy, fluoroscopy, intervention, phantoms, protective device, radiation exposure

## Abstract

Adoption of interventional endoscopic procedures is increasing with increasing prevalence of diseases. However, medical radiation exposure is concerning; therefore, radiation protection for medical staff is important. However, there is limited information on the usefulness of an additional lead shielding device during interventional endoscopic procedures. Therefore, we aimed to determine whether an additional lead shielding device protects medical staff from radiation.

An X-ray unit (CUREVISTA; Hitachi Medical Systems, Tokyo, Japan) with an over-couch X-ray system was used. Fluoroscopy-associated scattered radiation was measured using a water phantom placed at the locations of the endoscopist, assistant, nurse, and clinical engineer. For each location, measurements were performed at the gonad and thyroid gland/eye levels. Comparisons were performed between with and without the additional lead shielding device and with and without a gap in the shielding device. Additionally, a clinical study was performed with 27 endoscopic retrograde cholangiopancreatography procedures.

The scattered radiation dose was lower with than without additional lead shielding at all medical staff locations and decreased by 84.7%, 82.8%, 78.2%, and 83.7%, respectively, at the gonad level and by 89.2%, 86.4%, 91.2%, and 87.0%, respectively, at the thyroid gland/eye level. Additionally, the scattered radiation dose was lower without than with a gap in the shielding device at all locations.

An additional lead shielding device could protect medical staff from radiation during interventional endoscopic procedures. However, gaps in protective equipment reduce effectiveness and should be eliminated.

## 1 Introduction

The adoption of interventional endoscopic procedures, including endoscopic retrograde cholangiopancreatography (ERCP) and endoscopic ultrasonography (EUS)-guided intervention, has been increasing, along with an increase in the prevalence of diseases such as bile duct carcinoma and pancreatic carcinoma. ERCP and EUS-guided intervention have various therapeutic applications, and they are relatively less invasive than previously used surgical procedures. However, medical radiation exposure is of great concern because of its increasing frequency and potential carcinogenic effects.^[1–4]^ Therefore, radiation protection for medical staff is important.^[5–9]^

Several factors influence the radiation exposure of medical staff during interventional endoscopic procedures, and one of these factors is the use of a personal protective equipment or radiation protection shields.^[10]^ However, the use of these protection devices often leads to a lack of awareness regarding radiation hazards and discomfort. In our hospital, approximately 450 endoscopic procedures involving fluoroscopy, including ERCP, choledocholithotomy, bile duct and pancreatic duct stent insertion, double-balloon ERCP, and EUS drainage, are performed annually. Because of this high number of procedures, radiation exposure among medical staff is significant. The International Commission on Radiological Protection (ICRP) has recommended a maximum safe limit for the effective dose of 20 mSv/yr (averaged over a defined 5-year period with no single year exceeding 50 mSv) for the whole body as well as for the eye.^[11]^ The European Society of Digestive Endoscopy also recommended 20 mSv/yr.^[10]^

**Figure 1:**
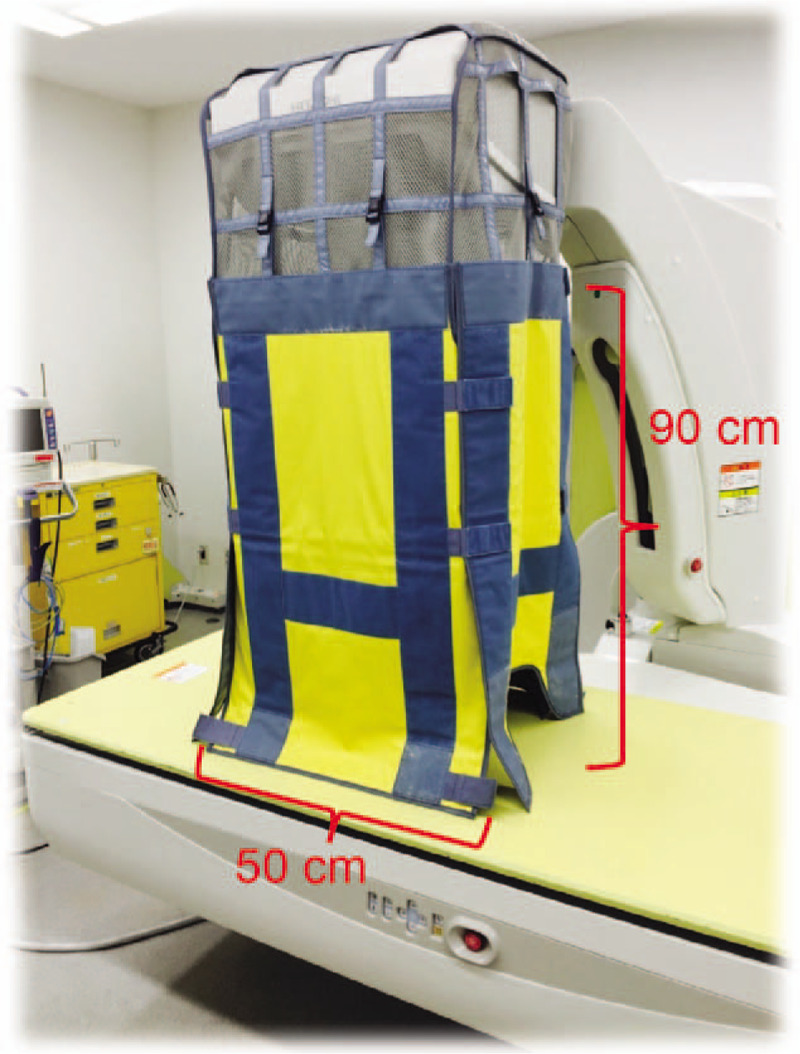
The CUREVISTA imaging device (Hitachi Medical Systems, Tokyo, Japan) with additional lead shielding. The front and back of the additional lead shielding device are 90 × 50 cm, and the 2 sides are 90 × 50 cm.

Considering this background, we have been using scattered radiation protection cloth during endoscopic procedures (Fig. 1). Although there have been reports on the use of additional lead shielding devices during cardiac catheterization procedures,^[12–15]^ few studies have examined scattered radiation doses received by medical staff during endoscopic procedures, including ERCP.^[2–4,16]^ Therefore, the present study aimed to compare radiation doses during endoscopic procedures before and after the installation of an additional lead shielding device, attached to the operating room table, and to determine whether this additional lead shielding device can further protect endoscopists and other medical staff, especially nurses, from radiation.

## 2 Methods

### 2.1 X-ray unit and additional lead shielding device during interventional endoscopy

**Figure 2:**
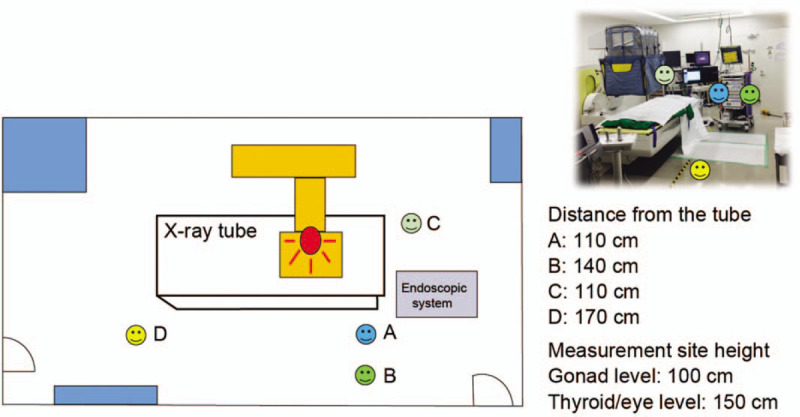
Layout for the phantom study and measurement of medical staff radiation exposure. Locations of the medical staff relative to the X-ray tube during the procedure are indicated as follows: (A) endoscopist's location; (B) assistant's location; (C) nurse's location; and (D) clinical engineer's location.

An X-ray unit (CUREVISTA; Hitachi Medical Systems, Tokyo, Japan) with an over-couch X-ray system was used. The X-ray unit has a width of 210 cm, height of 265.9 cm (maximum at the time of falling), depth of 215 cm, and mass of 1950 kg. The control system of this equipment was set to X-ray irradiation at 55 kV and 1.0 mA. The fluoroscopy mode was set as pulsed fluoroscopy, with a rate of 30 frames/s. Since 2012, we have been using an additional lead shielding device (Hitachi Medical Systems; 0.125 mm lead equivalent), which includes 4 lead shielding sheets (the front and back are 90 × 50 cm, and the 2 sides are 90 × 50 cm). It is hung down from the cine camera to the surface of the operating table during interventional endoscopy (Fig. 1). The upper part of the shield is made of mesh (no lead shield). The locations of each staff member relative to the X-ray tube during interventional endoscopy are presented in Figure 2.

### 2.2 Phantom study

**Figure 3:**
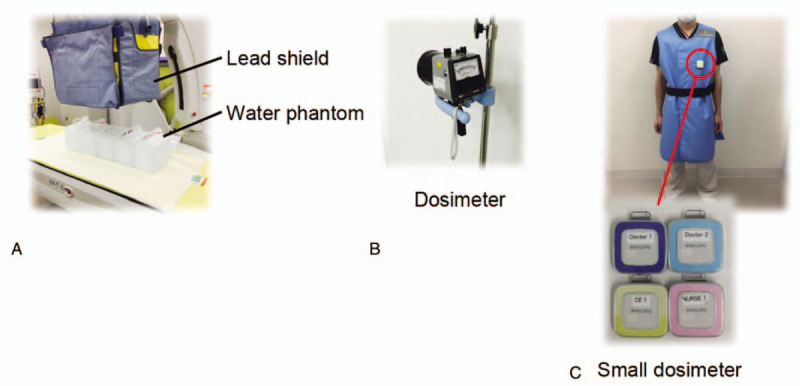
Devices used in the present study. In the phantom study, we used a water phantom (A) and dosimeter (ICS-301, Hitachi-Aloka Medical, Ltd., Tokyo, Japan) (B). In the clinical study, we used a small dosimeter (Dose Aware, Philips, Tokyo, Japan) (C).

Fluoroscopy-associated scattered radiation was measured using a water phantom. The water phantom included 4 acrylic bottles (width: 20 cm; height: 20 cm; depth: 15 cm) and was placed in the longitudinal direction on a transparent table considering the expected location of a patient during an interventional endoscopic procedure (Fig. 3A).

**Figure 4:**
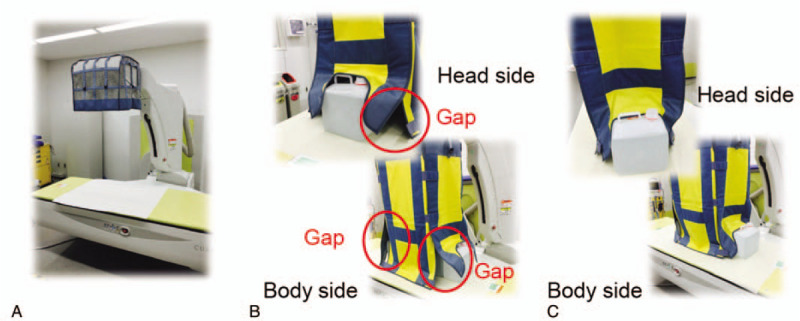
Measurement conditions. Measurements were performed without the additional lead shielding device (A), with the additional lead shielding device and with a gap in shielding (B), and with the additional lead shielding device and without a gap in shielding (C). The upper part of the device is made of mesh.

Data were obtained from the locations of the endoscopist, assistant, nurse, and clinical engineer. For each location, measurements were made at 2 heights above the floor (100 cm, corresponding to the approximate position of the gonads, and 150 cm, corresponding to the approximate position of the thyroid gland/eyes). These measurements were performed using a radiation survey dosimeter (ICS-301; Hitachi-Aloka Medical, Ltd., Tokyo, Japan), with a measurement range of 1 μSv/h to 300 mSv/h (Fig. 3B). The measurement conditions were as follows:

(1) without the additional lead shielding device (Fig. 4A);
(2) with the additional lead shielding device and with a gap in shielding (Fig. 4B); and
(3) with the additional lead shielding device and without a gap in shielding (Fig. 4C).

### 2.3 Exposure dose measurements of the operator and other staff members

The X-ray equipment settings and measurement locations were the same as those used in the phantom study, but the height evaluation was only at the left chest with a small dosimeter (Dose Aware, Philips, Tokyo, Japan) (Fig. 3C). Comparisons of the exposure doses were performed at the locations of the endoscopist, assistant, nurse, and clinical engineer during 27 ERCP procedures from April 2017 to September 2017.

### 2.4 Statistical analysis and patient and public involvement

The statistical method used was Tukey test, and the significance level was 5%. All statistical analyses were conducted using R software (R for Windows V.3.5.1; The R Foundation for Statistical Computing, Vienna, Austria).

This research was the observational study that performed without patient involvement. Patients were not invited to comment on the study design and were not consulted to develop patient-relevant outcomes or interpret the results. Patients were also not invited to contribute to the writing or editing of this document for readability or accuracy. This was the part of the in-hospital survey for the health of medical staffs who expose the radiation. Thus, it was determined that ethical approval was not necessary.

## 3 Results

### 3.1 Phantom study

**Figure 5:**
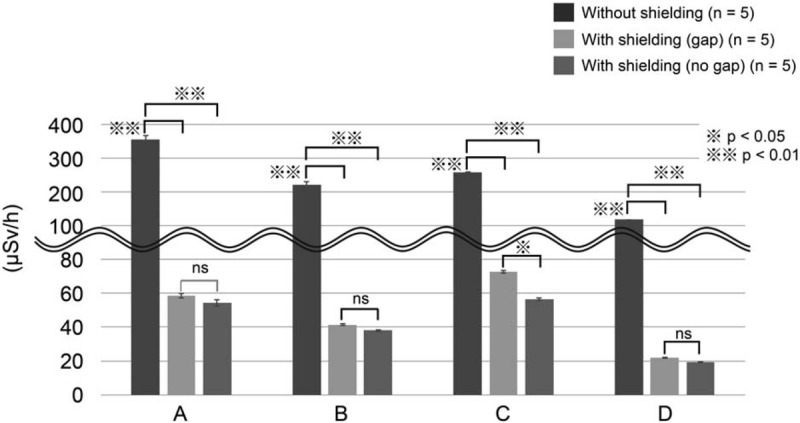
Radiation exposure (gonad level) at the medical staff locations in the phantom study. Radiation exposure at the gonad level was assessed at the following locations: (A) endoscopist's location; (B) assistant's location; (C) nurse's location; and (D) clinical engineer's location. The measurement conditions were as follows: without the additional lead shielding device, with the additional lead shielding device and with a gap in shielding, and with the additional lead shielding device and without a gap in shielding.

The scattered radiation doses at 100 cm above the floor (gonad level) with and without the additional lead shielding device are shown in Figure 5 and Table 1. At the endoscopist's location, the doses without and with the additional lead shielding device were 356.0 ± 2.4 μSv/h and 54.6 ± 1.8 μSv/h, respectively; thus, the scattered radiation dose decreased by 84.7% with shielding. At the assistant's location, the doses without and with the additional lead shielding device were 222.0 ± 2.0 μSv/h and 38.2 ± 0.37 μSv/h, respectively; thus, the scattered radiation dose decreased by 82.8% with shielding. At the nurse's location, the doses without and with the additional lead shielding device were 260.0 μSv/h and 56.8 ± 0.58 μSv/h, respectively; thus, the scattered radiation dose decreased by 78.2% with shielding. At the clinical engineer's location, the doses without and with the additional lead shielding device were 120.0 μSv/h and 19.6 ± 0.25 μSv/h, respectively; thus, the scattered radiation dose decreased by 83.7% with shielding.

**Table 1:**
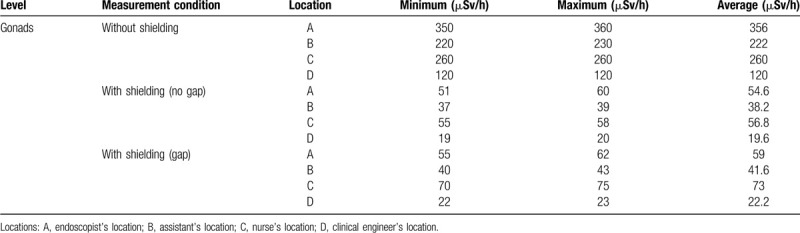
Radiation exposure (gonad level) in the phantom study.

We also compared the scattered radiation doses between with and without a gap in the additional lead shielding device. The scattered radiation doses at the gonad level with and without a gap in the additional lead shielding device are shown in Figure 5 and Table 1. At the endoscopist's location, the doses with and without a gap were 59.0 ± 1.22 μSv/h and 54.6 ± 1.83 μSv/h, respectively; thus, the scattered radiation doses were similar. At the assistant's location, the doses with and without a gap were 41.6 ± 0.51 μSv/h and 38.2 ± 0.37 μSv/h, respectively; thus, the scattered radiation doses were similar. At the nurse's location, the doses with and without a gap were 73.0 ± 0.95 μSv/h and 56.8 ± 0.58 μSv/h, respectively; thus, the scattered radiation dose decreased by 22.2% without a gap. At the clinical engineer's location, the doses with and without a gap were 22.2 ± 0.2 μSv/h and 19.6 ± 0.25 μSv/h, respectively; thus, the scattered radiation doses were similar.

**Figure 6:**
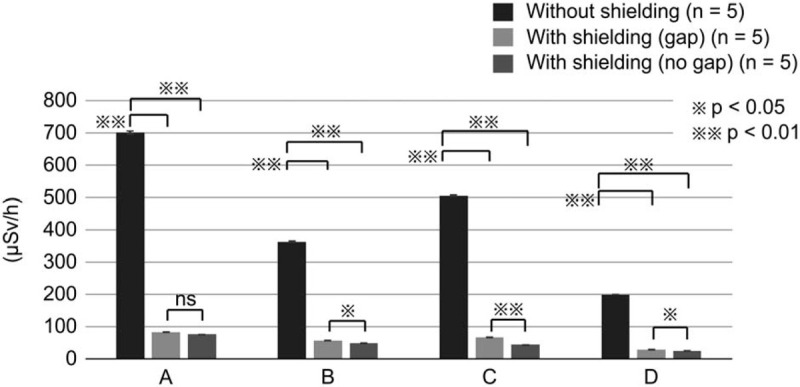
Radiation exposure (thyroid gland/eye level) at the medical staff locations in the phantom study. Radiation exposure at the thyroid gland/eye level was assessed at the following locations: (A) endoscopist's location; (B) assistant's location; (C) nurse's location; and (D) clinical engineer's location. The measurement conditions were as follows: without the additional lead shielding device, with the additional lead shielding device and with a gap in shielding, and with the additional lead shielding device and without a gap in shielding.

The scattered radiation doses at 150 cm above the floor (thyroid gland/eye level) with and without the additional lead shielding device are shown in Figure 6 and Table 2. At the endoscopist's location, the doses without and with the additional lead shielding device were 702.0 ± 4.9 μSv/h and 76.2 ± 0.7 μSv/h, respectively; thus, the scattered radiation dose decreased by 89.2% with shielding. At the assistant's location, the doses without and with the additional lead shielding device were 364 ± 2.4 μSv/h and 49.6 ± 0.2 μSv/h, respectively; thus, the scattered radiation dose decreased by 86.4% with shielding. At the nurse's location, the doses without and with the additional lead shielding device were 506 ± 2.4 μSv/h and 44.4 ± 0.4 μSv/h, respectively; thus, the scattered radiation dose decreased by 91.2% with shielding. At the clinical engineer's location, the doses without and with the additional lead shielding device were 200 ± 0 μSv/h and 26 ± 0.4 μSv/h, respectively; thus, the scattered radiation dose decreased by 87.0% with shielding.

**Table 2:**
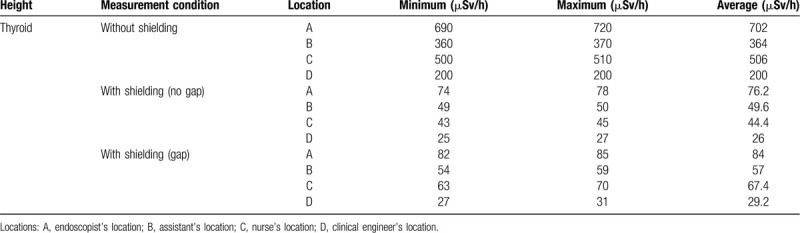
Radiation exposure (thyroid gland/eye level) in the phantom study.

The scattered radiation doses at the thyroid gland/eye level with and without a gap in the additional lead shielding device are shown in Figure 6 and Table 2. At the endoscopist's location, the doses with and without a gap were 84.0 ± 0.55 μSv/h and 76.2 ± 0.66 μSv/h, respectively; thus, the scattered radiation doses were similar. At the assistant's location, the doses with and without a gap were 57.0 ± 0.89 μSv/h and 49.6 ± 0.24 μSv/h, respectively; thus, the scattered radiation dose decreased by 13% without a gap. At the nurse's location, the doses with and without a gap were 67.4 ± 0.17 μSv/h and 44.4 ± 0.40 μSv/h, respectively; thus, the scattered radiation dose decreased by 34% without a gap. At the clinical engineer's location, the doses with and without a gap were 29.2 ± 0.73 μSv/h and 26.0 ± 0.45 μSv/h, respectively; thus, the scattered radiation dose decreased by 11% without a gap.

### 3.2 Exposure doses of the operator and other staff members

**Figure 7:**
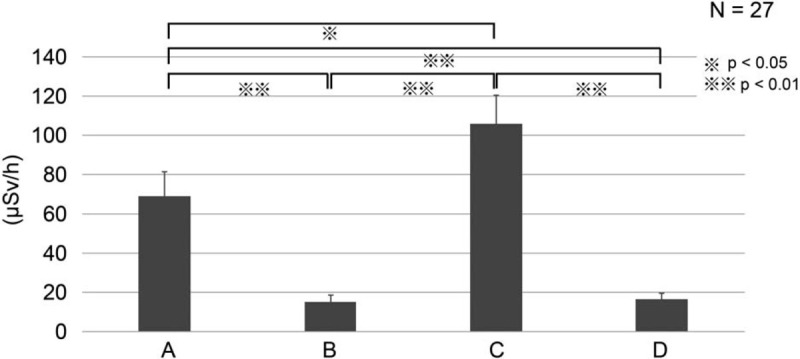
Radiation exposure at the medical staff locations in the clinical study. Radiation exposure was assessed at the following locations: (A) endoscopist's location; (B) assistant's location; (C) nurse's location; and (D) clinical engineer's location.

With regard to the 27 ERCP procedures in which a shielding device was used, the mean procedure time was 34.9 minutes and the mean fluoroscopy time was 22.3 minutes (Table 3). The exposure doses of the endoscopist and other staff members are shown in Figure 7. At the endoscopist's location, the dose was 28.8 ± 34.5 μSv/procedure (69.0 ± 12.4 μSv/h). At the assistant's location, the dose was 6.9 ± 7.0 μSv/procedure (15.2 ± 3.5 μSv/h). At the nurse's location, the dose was 47.9 ± 45.1 μSv/procedure (105.8 ± 14.9 μSv/h). At the clinical engineer's location, the dose was 7.4 ± 10.2 μSv/procedure (16.7 ± 2.9 μSv/h). Thus, the dose was significantly higher at the nurse's location than at the other 3 members’ locations (*P* < .05) (Fig. 7).

**Table 3:**
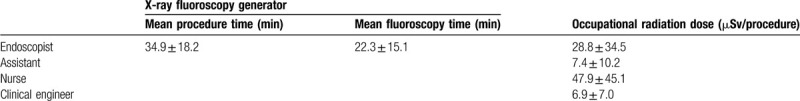
Radiation exposure in the clinical study.

## 4 Discussion

The present study found that the additional lead shielding device greatly decreased the scattered radiation dose at all locations of medical staff and that the absence of a gap in the shielding device tended to decrease the scattered radiation dose. To the best of our knowledge, this is the first study to examine not only the usefulness of the additional lead shielding device but also the scattered radiation dose from shielding gaps.

Recently, the adoption of interventional endoscopic procedures, such as ERCP, has been increasing because of their less invasiveness. As a result, radiation exposure among medical staff will increase. The exposure dose should not exceed the ICRP dose limit of 100 mSv/5 years.^[9,17–19]^ Additionally, the regulatory effective dose limit (20 mSv/yr averaged over 5 consecutive years, 100 mSv over 5 years, and 50 mSv in 1 year) is used to ensure that the risk of occurrence of stochastic effects is maintained within acceptable levels. To prevent the occurrence of stochastic effects, the radiation dose should be as low as reasonably possible while ensuring that the procedure is diagnostically useful and efficiently performed. Therefore, radiation protection for medical staff, including physicians performing interventional endoscopy, is particularly important.^[20]^

The extent of radiation exposure among medical staff is influenced by various factors, such as the environment of the endoscopy unit, distance between the medical staff and radiation source or patient, type of X-ray system (over-couch, under-couch, or mobile C-arm unit), fluoroscopy parameters (use of pulsed rather than continuous fluoroscopy, use of lower frame rates of fluoroscopy, number of radiographs, use of X-ray beam collimation, or use of low magnification), and use of protective equipment.^[10,21–24]^ Moreover, the fluoroscopy time is influenced by various factors, such as the difficulty of the procedure,^[25,26]^ proficiency levels of the endoscopist and assistant,^[6]^ education and awareness of radiation protection,^[27,28]^ and number of interventional endoscopic procedures during a specific period. Our institution uses an over-couch X-ray system associated with high radiation exposure at the endoscopist's thyroid gland and eye levels, and the dose might exceed the ICRP limit.^[20]^

Various approaches have been adopted to protect medical staff from radiation during interventional endoscopic procedures, and the use of individual protective equipment is one of the approaches.^[10]^ However, individual protective equipment does not cover the entire body. Additionally, its high weight limits movement, and it is sometimes avoided owing to discomfort. Therefore, our institution has been using scattered radiation protection cloth to protect medical staff from radiation during interventional endoscopic procedures.

There are several reports on the use of scattered radiation protection cloth for protecting medical staff from radiation during interventional endoscopic procedures. Minami et al used radiation-attenuating curtains mounted on the X-ray tube.^[16]^ Among endoscopists, the mean radiation doses per procedure with the protective curtains and without them were 42.6 μSv and 340.9 μSv, respectively. Additionally, Morishima et al reported that among endoscopists, the mean radiation doses per procedure with and without the additional lead shielding device were 31.9 μSv and 87.8 μSv, respectively (dose reduction of 63.7%).^[2]^ The mean radiation dose per procedure at the endoscopist's location was lower in our study (28.8 μSv) than in previous studies. Additionally, our phantom study revealed the usefulness of the additional lead shielding device. The amount of radiation drastically reduced with the use of the protective device, and at the endoscopist's location (gonad level), the dose significantly reduced from 356.0 μSv/h (without the device) to 54.6 μSv/h (with the device) (reduction of approximately 85%) (*P* < .01).

However, there is concern regarding radiation leakage from gaps in the protective equipment owing to differences in patient body type and changes in posture. The mean occupational radiation dose per interventional endoscopic procedure was the highest among nurses (47.9 μSv, which was equal to 105.1 μSv/h), and this dose was higher than that obtained in the phantom study. In the phantom study, at the nurse's location, the mean doses with and without a shielding gap were 73.0 μSv/h and 56.8 μSv/h, respectively, and these findings might be associated with the fact that nurses monitor the general condition of the patient, including the respiratory condition, at the head side of the patient in our hospital. Minami et al mentioned that a disadvantage of additional protective equipment was that it could block the monitoring of the patient.^[16]^ The nurse's location in our hospital is important for monitoring the general condition of a patient during a procedure. However, the nurse's location is most likely to be influenced by the movement of the patient and shielding gaps in protective equipment, and elimination of the shielding gaps might reduce radiation exposure among nurses. On the other hand, the radiation dose of the endoscopists is not influenced by the shielding gaps so much despite of closest location. The reason is that the location of the endoscopists is right in front of the shielding device.

The present study has some limitations. First, this was a single-center study, and data were obtained from only 2 endoscopists, 1 nurse, and 1 clinical engineer. Second, the study was conducted for a relatively short period of 6 months, and the number of ERCPs was small. Finally, radiation exposure among the medical staff was calculated without considering the radiation exposure time and patient body weight. Although the survey meter used for measurements was regularly calibrated, there might have been variations in the measurements.

In conclusion, an additional lead shielding device can protect medical staff from radiation exposure during interventional endoscopic procedures, even if there is radiation leakage from shielding gaps. Elimination of the shielding gaps of protective equipment can cause an approximately 90% reduction in exposure to scattered radiation.

## Acknowledgments

The authors thank Mr. Hiroaki Maki and Mr. Tsuyoshi Yamada, who are radiological technicians at Mie University Hospital, for their invaluable assistance.

## Author contributions

Yusuke Saimyo and Reiko Yamada carried out the studies and drafted the manuscript. Yusuke Saimyo participated in the design of the study and performed the statistical analysis. Reiko Yamada conceived of the study, and participated in its design and coordination. Kyosuke Tanaka, Aiji Hattori, Yuhei Umeda, Naoki Kuroda, Junya Tsuboi, Yasuhiko Hamada, Yoshiyuki Takei helped to draft the manuscript. All authors read and approved the final manuscript.
